# Retrieval of a fractured PICC fragment following umbilical venous insertion in a preterm infant: a case report

**DOI:** 10.3389/fped.2026.1724088

**Published:** 2026-04-13

**Authors:** Shaolong Niu, Yunchao Sun, Jianmin Wang, Liangshuo Wan, Ziyuan Zhang, Xin Zhang, Zening Ma

**Affiliations:** 1 Graduate School, Hebei University of Chinese Medicine, Shijiazhuang, Hebei, China; 2 Department of Peripheral Vascular Surgery, Hebei Hospital of Traditional Chinese Medicine, Shijiazhuang, Hebei, China

**Keywords:** catheter fracture, endovascular, retrieval, intracardiac foreign body, neonatal interventional radiology, peripherally inserted cenral catheter (off-label umbilical insertion), preterm infant

## Abstract

**Background:**

Due to the immaturity of their organs, preterm infants often require central venous access to ensure effective treatment and nutritional support. Although umbilical vein catheterization is a commonly used method, rare but serious complications such as catheter fracture and migration can occur during the procedure, posing significant challenges and high risks to manage.

**Methods:**

This report describes a case of a male preterm infant with a gestational age of 32 weeks who required a peripherally inserted central catheter (PICC device inserted via the umbilical vein as an off-label approach) for critical care. During catheter removal, the catheter fractured and migrated to the right atrium. Given the high risk of surgical intervention, an endovascular approach via the femoral vein was chosen for foreign body retrieval. Under fluoroscopic guidance using a digital subtraction angiography (DSA) system without contrast, the fractured catheter segment was successfully removed using a multi-wire system and catheter pathway construction combined with a lumen snare system.

**Results:**

The procedure was performed smoothly, with stable vital signs observed throughout the operation and no perioperative arrhythmias. Postoperative imaging confirmed the absence of thrombus formation or residual catheter fragments, and the infant exhibited favorable recovery.

**Conclusion:**

This case demonstrates that minimally invasive endovascular retrieval is a safe and effective alternative for managing intracardiac foreign bodies in preterm infants, particularly those at high surgical risk. The refined technique without contrast agent use further enhances procedural safety for such high-risk neonates, offering valuable clinical insights for managing similar catheter-related complications.

## 1 Introduction

Preterm infants (neonates delivered before 37 weeks of gestation) typically exhibit low birth weight and poor physiological reserve due to insufficient intrauterine development, resulting in immature organ systems. This predisposes them to complications such as hypotension, neonatal respiratory distress syndrome (NRDS), and infections. Such infants often require prolonged intravenous access for medication administration and nutritional support to sustain life and ensure proper development (1). Consequently, establishing safe and reliable venous access remains a critical aspect of clinical management. Currently, umbilical vein catheterization (UVC) and PICC are the preferred choices in neonatal intensive care units, each with distinct advantages and limitations (2). In the immediate postnatal period, the umbilical vein maintains a relatively large diameter, making UVC technically easier than PICC placement; however, PICCs are generally intended for longer-term central venous access when placed via appropriate peripheral venous routes (3). In clinical practice, when umbilical or peripheral venous access proves suboptimal or when extended central venous access is required, the insertion of a PICC device via the umbilical vein may be considered as a provisional, off-label measure after thorough clinical evaluation (4). In extremely preterm infants with limited peripheral venous access, clinicians may occasionally consider alternative access routes in emergency situations. Although PICCs are not specifically designed for umbilical insertion, several reports have described temporary off-label use when conventional vascular access cannot be established (5). In such circumstances, careful monitoring and imaging guidance are essential to minimize procedural risks. This report presents a case of a 32-week preterm infant who experienced catheter fracture involving a PICC device following off-label umbilical vein insertion, with subsequent successful retrieval under fluoroscopic guidance using a DSA system without contrast.

## 2 Clinical data

### 2.1 Basic information

The patient was a male preterm infant born at 32 weeks of gestation via emergency cesarean section on May 18, 2025, at a local hospital, with a birth weight of 760 g. There was no known history of congenital anomalies or hereditary diseases, and no maternal complications were reported during pregnancy. The infant had no prior history of vascular interventional procedures before catheter placement. No psychosocial or genetic abnormalities were identified. At birth, the infant exhibited weak spontaneous respiration and hypotonia. Immediate resuscitative measures were initiated, including thermal support, endotracheal intubation, and positive pressure ventilation with a resuscitation bag. Once stabilized, he was transferred to our hospital on May 19, 2025, under mechanical ventilation support. On admission, urgent arterial blood gas analysis revealed the following: pH 7.19, PCO₂ 73.7 mmHg, PO₂ 49 mmHg, lactate 3.2 mmol/L, and base excess (BE) −0.6 mmol/L. Capillary blood glucose was 2.5 mmol/L. Complete blood count showed: white blood cell count (WBC) 7.69 × 10⁹/L, hemoglobin (HGB) 125 g/L, and platelet count (PLT) 16 × 10⁹/L. Coagulation profile demonstrated significant abnormalities: prothrombin time (PT) 19.3 s, international normalized ratio (INR) 1.75, activated partial thromboplastin time (APTT) 74.4 s, thrombin time (TT) 23.6 s, plasma fibrinogen (Fib) 0.87 g/L, and antithrombin III (AT-III) 21%. Apgar scores were 7 at 1 min and 8 at both 5 and 10 min. The initial diagnoses included neonatal respiratory failure, hyperlactatemia, and coagulation dysfunction.

### 2.2 Treatment course and follow-up

**Figure 1:**
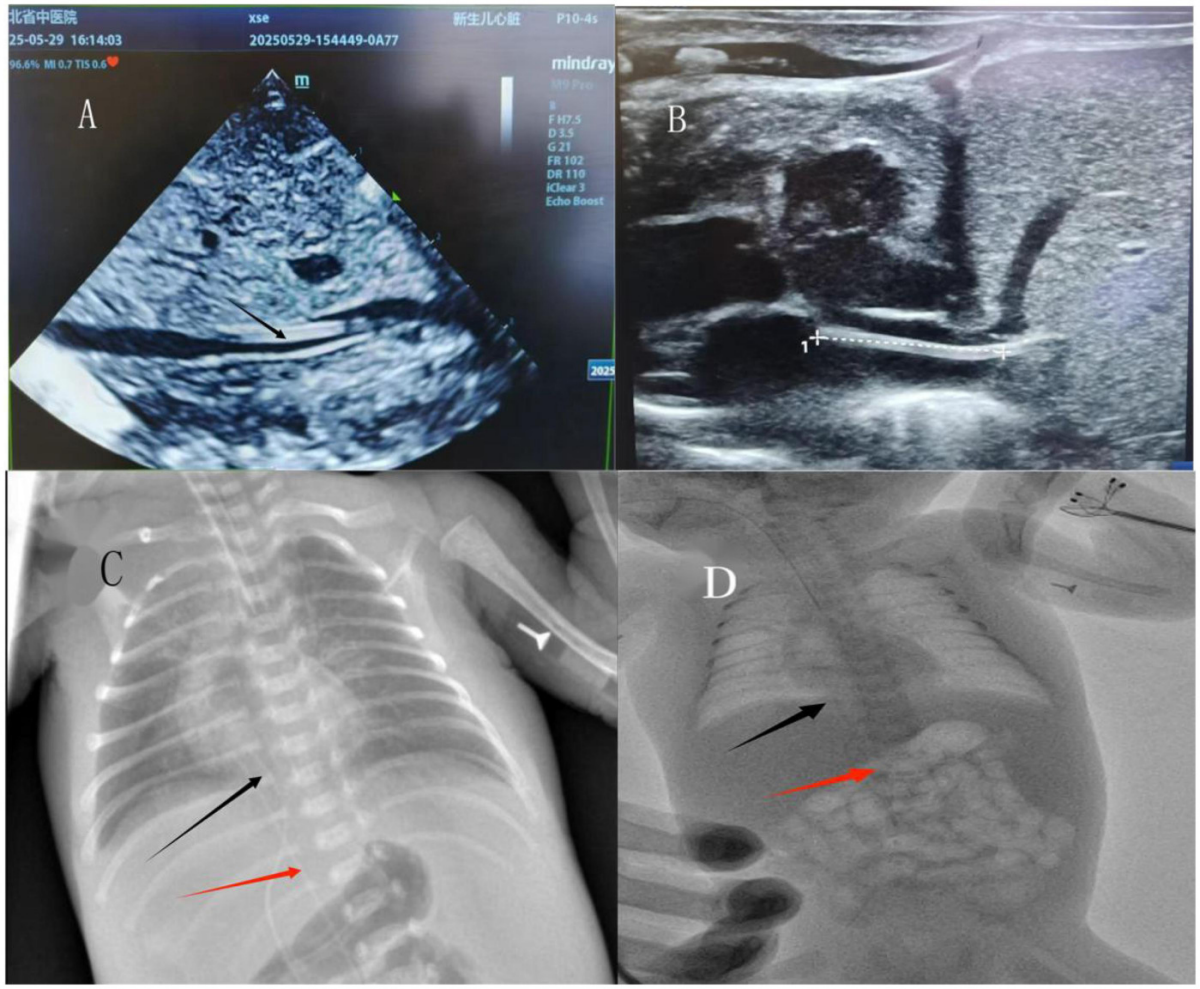
Preoperative imaging findings: **(A, B)** cardiac echocardiography. **(C)** Preoperative bedside x-ray (red arrow: the femoral venous PICC catheter, black arrow: the residual segment of the umbilical venous PICC catheter). **(D)** Preoperative fluoroscopic imaging (red arrow: the femoral venous PICC catheter, black arrow: the residual segment of the umbilical venous PICC catheter).

To establish reliable central venous access, umbilical vein catheterization was initially attempted. However, due to the narrow caliber of the umbilical vein and difficulty in identifying the femoral vein, the procedure was unsuccessful. As an emergency alternative, a peripherally inserted central catheter (PICC device) was inserted via the umbilical vein as an off-label measure to achieve temporary central venous access. On May 29, 2025, under ultrasound guidance, a new PICC line was successfully inserted via the right femoral vein. Simultaneously, the previously placed PICC device inserted via the umbilical vein was withdrawn. During the removal process, catheter fracture occurred. An emergent echocardiographic evaluation (Figures 1A, B) revealed echogenic catheter remnants extending from the umbilical vein through the hepatic vein into the inferior vena cava (IVC), with the proximal tip located approximately 10 mm from the umbilical stump and the distal end within the IVC. Bedside radiography (Figure 1C) demonstrated a linear hyperdense structure overlapping the spine at the level of the sixth posterior rib, consistent with a retained intravascular catheter fragment. These findings confirmed the presence of a single retained intravascular catheter fragment extending from the umbilical vein into the inferior vena cava. Based on the clinical course and imaging findings, a diagnosis of intravascular retention of a fractured catheter fragment was established. Differential diagnoses such as thrombus or calcified vascular structures were considered unlikely based on the linear morphology and imaging characteristics. The primary diagnostic challenge was accurate localization of the fragment in a preterm infant with small vessel caliber and limited tolerance for prolonged imaging. An urgent multidisciplinary consultation was conducted, involving pediatric surgery, cardiothoracic surgery, interventional radiology, and ultrasonography teams. Although the proximal tip of the fragment was close to the umbilical stump, surgical exploration was considered high risk due to severe coagulation dysfunction and the potential for significant bleeding in this preterm infant. Following comprehensive risk-benefit assessment, image-guided endovascular retrieval was recommended. On physical examination prior to intervention, the infant was hemodynamically stable, with normal cardiac auscultation and no signs of acute distress.

#### 2.2.1 Surgical procedure

**Figure 2:**
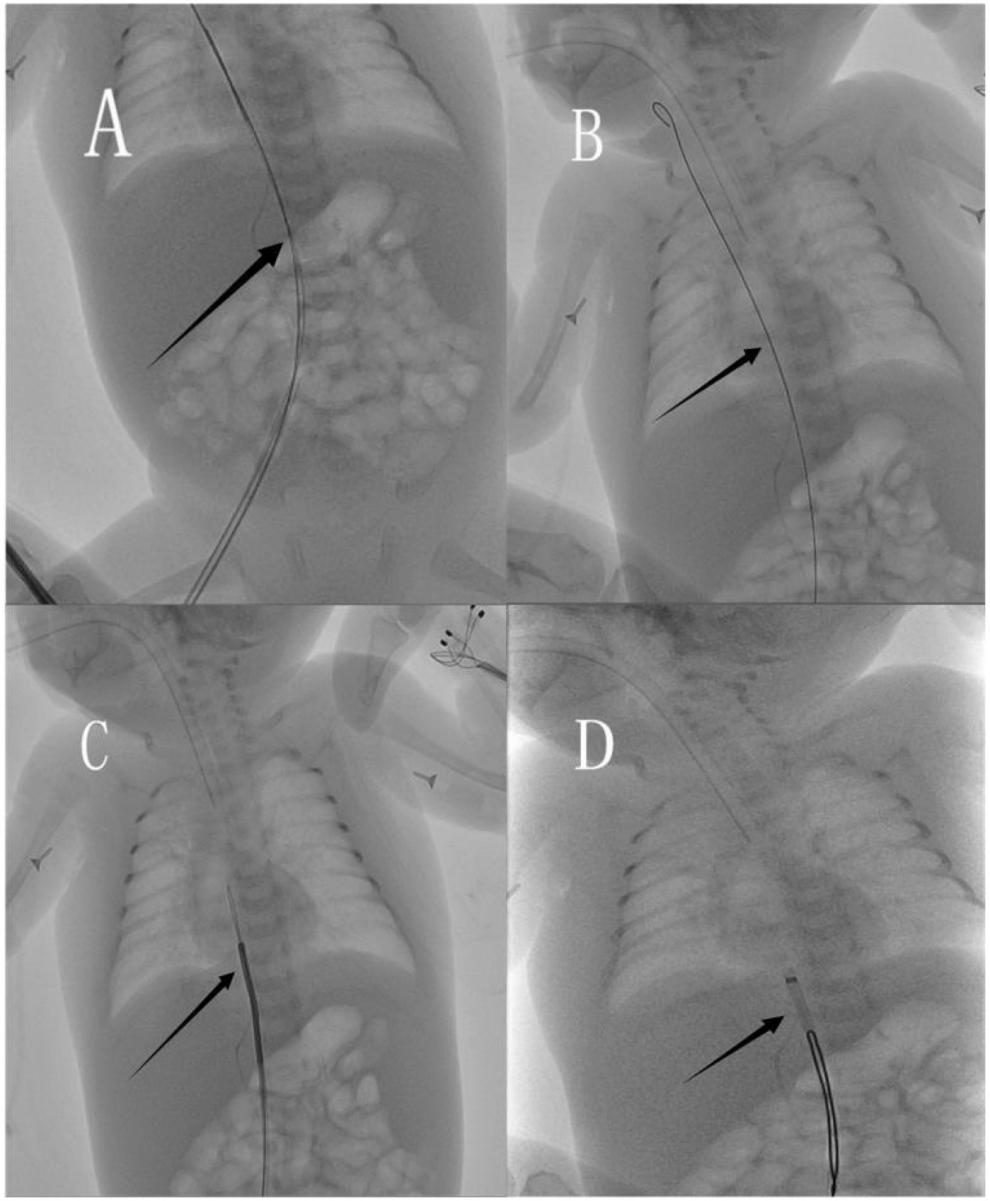
Intraoperative fluoroscopic images during endovascular retrieval: **(A)** guidewire–catheter system positioned in the right atrium. **(B)** Formation of a stable intravascular pathway. **(C)** Loop-guidewire attempt to engage the distal fragment. **(D)** Snare capture of the catheter fragment.

**Figure 3:**
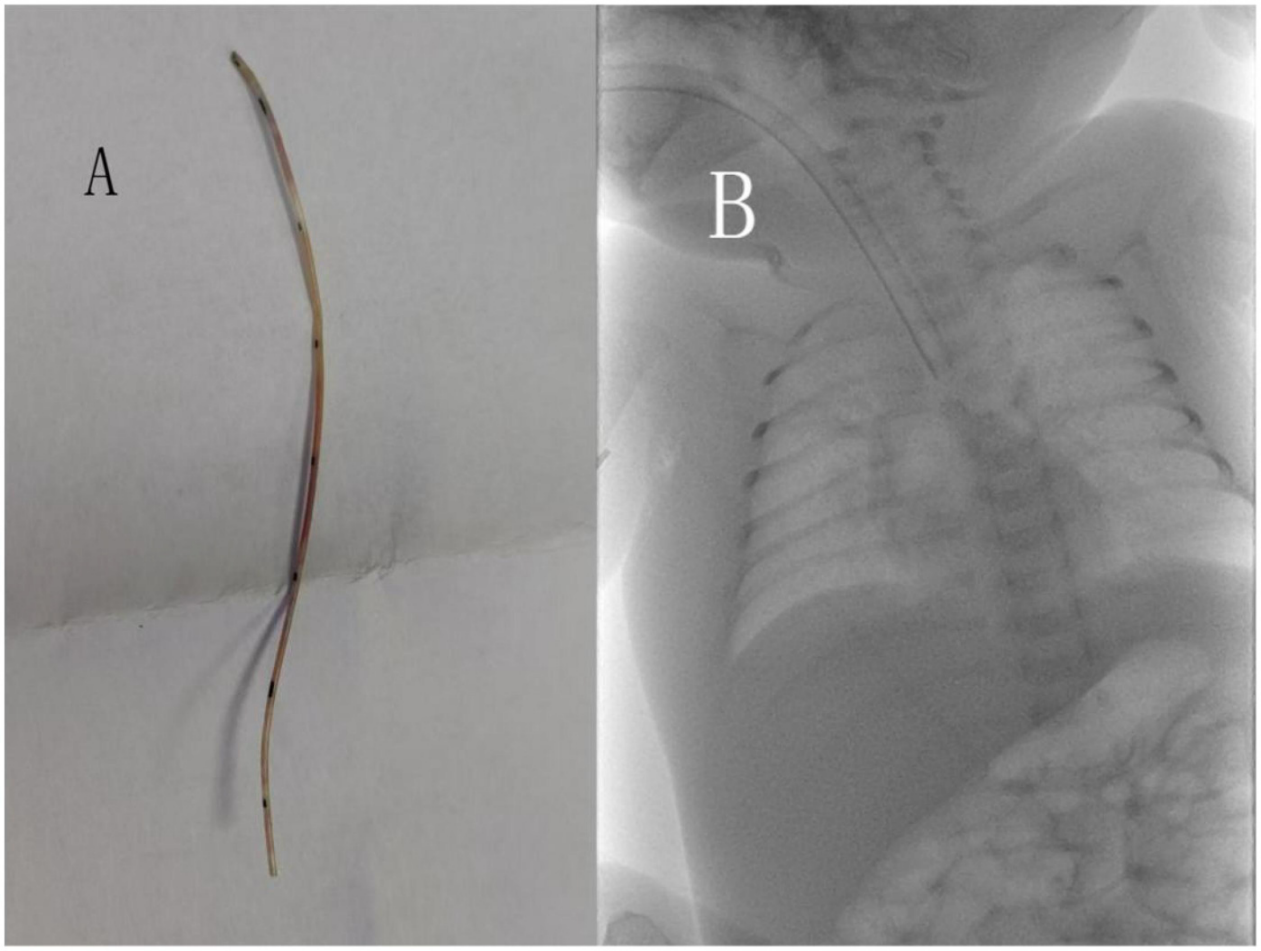
Postoperative findings: **(A)** retrieved catheter fragment. **(B)** Final fluoroscopic image.

Under fluoroscopic guidance using a DSA system without contrast, the position of the retained catheter fragment was reconfirmed (Figure 1D). Preoperative echocardiography and the radiopaque nature of the catheter fragment allowed clear visualization and localization under real-time fluoroscopy, enabling device manipulation without the need for angiographic contrast injection. Vascular access was obtained via the right femoral vein. A stepwise endovascular retrieval strategy was adopted to minimize vascular trauma in this preterm infant. Initially, a guidewire-assisted approach was attempted to engage the distal end of the foreign body. Sequential guidewire exchanges were performed to establish a stable intravascular pathway (Figures 2A, B). A U-shaped guidewire loop was carefully formed and advanced toward the distal end of the retained catheter fragment in an attempt to retrieve it without the use of a large-caliber sheath (Figure 2C). However, this maneuver was unsuccessful. Subsequently, a snare retrieval system was introduced through a 6F sheath under continuous fluoroscopic guidance. The snare was manipulated to encircle and securely capture the distal end of the catheter fragment (Figure 2D). The snare and sheath were withdrawn together, resulting in successful extraction of an approximately 7.5-cm PICC segment (Figure 3A). Final fluoroscopic examination confirmed complete removal of the foreign body, with no residual fragments in the cardiac chambers or abdominal vessels (Figure 3B). Manual compression was applied to the femoral puncture site, followed by sterile dressing. The infant remained hemodynamically stable throughout the procedure, with no arrhythmias or procedural complications. Notably, no contrast agent was used during the entire intervention. Furthermore, no general anesthesia or additional sedation was administered during the procedure. The intervention was performed under continuous electrocardiographic monitoring, with real-time supervision by neonatology, pediatric surgery, and critical care teams.

#### 2.2.2 Follow-up

At one-week follow-up, the puncture site in the right groin was healing well, with normal skin appearance and no signs of exudation or mass formation. The patient's parents expressed relief following successful retrieval of the catheter fragment and were satisfied with the minimally invasive management strategy, particularly given the avoidance of open surgery and contrast agents. (The specific clinical events and management timeline are presented in Table 1).

**Table 1:** Timeline of clinical events and management.

Date (2025)	Clinical event
May 18	Male preterm infant born at 32 weeks of gestation via emergency cesarean section at a local hospital; birth weight 760 g.
May 19	Attempted umbilical vein catheterization for central venous access; procedure unsuccessful due to narrow umbilical vein caliber and difficulty identifying suitable peripheral veins. As an emergency alternative, a peripherally inserted central catheter (PICC) was placed via the umbilical vein under close monitoring.
May 29	A new PICC line was successfully inserted via the right femoral vein under ultrasound guidance; removal of the previously placed umbilical PICC was attempted, during which catheter fracture occurred.
May 29	Emergency echocardiography and bedside radiography revealed a retained intravascular catheter fragment extending from the umbilical vein into the inferior vena cava.
May 29	Multidisciplinary consultation (neonatology, pediatric surgery, cardiothoracic surgery, interventional radiology, and ultrasonography); image-guided endovascular retrieval recommended.
May 29	Percutaneous intravascular retrieval performed via the right femoral vein under fluoroscopic guidance using a DSA system without contrast; foreign body successfully removed.
May 29	Post-procedural monitoring showed stable hemodynamics with no arrhythmia or immediate complications.
June 5	One-week follow-up: echocardiography confirmed complete removal of the catheter fragment; no thrombosis, bleeding, or cardiac structural abnormalities observed. The patient's parents reported satisfaction with the minimally invasive management.

## 3 Discussion

In recent years, complications related to central venous catheterization in neonates—particularly in extremely low birth weight (ELBW) and preterm infants—have garnered increasing attention in both domestic and international medical literature (6). Although the overall incidence of catheter-related intravascular foreign bodies remains low, such events are clinically significant due to their complex management and potential for serious outcomes. Establishing central venous access is essential for the resuscitation and nutritional support of critically ill neonates (7). Premature infants present unique challenges due to immature organ development, low body weight, small-caliber vessels, and reduced tissue elasticity. These anatomical and physiological factors increase the difficulty of peripheral venous cannulation and catheter advancement. As a result, UVC and PICC are widely used in neonatal intensive care settings to maintain reliable central venous access (8). UVC is often preferred in the early hours after birth, especially within the first 24 h, due to its ease of access and the direct anatomical pathway to central circulation. However, UVCs are generally suitable only for short-term use. The risk of complications such as bloodstream infection, thrombosis, and misplacement into the portal venous system increases with prolonged catheter duration (9). Compared with UVCs, PICCs are generally considered more suitable for long-term central venous access and may be associated with a lower risk of infection during prolonged use. However, in extremely preterm infants, PICC placement remains technically challenging and is associated with increased risks of vascular injury, malposition, and catheter migration (10). Studies have further shown that non-central positioning of PICC tips is associated with a significantly increased risk of complications, emphasizing the importance of intra-procedural imaging and optimal tip positioning (4). In specific clinical contexts where peripheral venous access is limited—such as in extremely premature neonates—some clinicians have adopted the off-label strategy of inserting PICCs via the umbilical vein. While this approach may temporarily achieve the dual goals of central access and extended catheter duration, it must be noted that PICCs are not originally designed for umbilical insertion. Resistance during advancement or improper withdrawal without imaging guidance may result in catheter fracture or migration, especially in fragile vascular environments (11, 12). Therefore, careful pre-procedural evaluation, device selection, and imaging-assisted manipulation are critical in minimizing such risks.

This study reports a case of catheter fracture and subsequent migration into the right atrium during off-label insertion of a PICC device via the umbilical vein in a preterm infant. Ultimately, the intravascular foreign body was successfully retrieved via a femoral venous approach under fluoroscopic guidance using a DSA system without contrast. The successful management of this case not only demonstrates the clinical value of precise interventional techniques in critical complications of preterm infants but also provides a minimally invasive reference approach for similar cases. In this case, the catheter fractured abruptly during the removal of a PICC device that had been inserted via the umbilical vein, with the residual segment migrating to the right atrium, forming a typical intracardiac foreign body. The fracture likely resulted from a combination of factors including the catheter's insertion angle, uneven tissue traction forces, and blind pulling without imaging guidance. This created a “shear-like fracture” at a tension concentration point along the catheter. This risk is especially pronounced in neonates, whose vessels have smaller diameters and more fragile tissue tension, where even slight force fluctuations can cause catheter tearing. Therefore, meticulous technique, standardized catheter removal procedures, and adequate operator experience are essential to minimize the risk of catheter fracture, particularly in preterm infants. Specifically, accurate assessment of catheter position and pathway before placement, as well as real-time imaging guidance during catheter removal using ultrasound or fluoroscopy, can help avoid forcibly pulling the catheter when it may be clamped by blood vessels or surrounding tissues, thereby preventing mechanical fracture. Additionally, operators should be familiar with the physical properties of different catheter materials, such as tensile strength, flexibility, and abrasion resistance, to select models and specifications better suited to the venous conditions of preterm infants (13, 14). Imaging data before and after catheter placement are crucial for preventing misdiagnosis and improper management of catheter fracture. Careful comparison of catheter insertion and removal lengths, combined with follow-up color Doppler ultrasound or x-ray examinations, can effectively identify retained fragments and related risks (15).

Intracardiac foreign bodies in neonates and preterm infants have been reported only sporadically in the literature, reflecting both their relative rarity and the technical challenges associated with management in this population. Intracardiac foreign bodies may lead to several potentially serious complications if not promptly managed. Reported adverse events include cardiac arrhythmias caused by mechanical irritation of the endocardium, thrombus formation around the retained fragment, systemic or pulmonary embolization, infective endocarditis, and, in rare cases, cardiac perforation (16). These complications are particularly concerning in preterm infants due to their limited physiological reserve and immature immune and coagulation systems. Therefore, once a catheter fragment is identified within the cardiovascular system, early removal is generally recommended whenever technically feasible. Early reports predominantly described open surgical removal via thoracotomy or laparotomy, which, although effective, are associated with substantial procedural trauma and high postoperative morbidity in extremely low birth weight and preterm infants (15). With advances in interventional techniques, percutaneous endovascular retrieval has increasingly been reported as a less invasive alternative and is now considered preferable when technically feasible. A review of published neonatal and pediatric cases indicates that most endovascular retrieval procedures for intracardiac foreign bodies have been performed under angiographic guidance with the use of contrast agents, which are typically employed to delineate vascular anatomy, confirm foreign body location, and guide device manipulation (17). Previous case reports have described successful percutaneous retrieval of catheter fragments in premature infants using snare systems, basket devices, or loop-wire techniques through femoral or jugular venous access. In most of these reports, angiographic contrast agents were routinely used to visualize vascular anatomy and guide device manipulation (18). However, in preterm infants with immature renal function, limited intravascular volume reserve, and increased hemodynamic vulnerability, contrast administration may introduce additional risks such as nephrotoxicity and fluid overload (19). Despite this, the feasibility of performing intracardiac foreign body retrieval without the use of contrast agents has rarely been specifically discussed in the existing literature. In this context, the present case demonstrates that successful retrieval can be achieved under contrast-free fluoroscopic guidance by integrating preoperative echocardiographic localization with real-time fluoroscopic monitoring and a stepwise endovascular strategy. This approach minimizes contrast-related risks while maintaining procedural safety and efficacy, thereby expanding the range of minimally invasive options for selected high-risk neonates. Rather than replacing conventional angiography-guided techniques, the contrast-free strategy illustrated in this case may serve as a complementary approach in carefully selected patients, particularly those with contraindications to contrast media or heightened susceptibility to renal or hemodynamic complications. Further accumulation of cases and comparative studies are warranted to better define its indications, limitations, and reproducibility in neonatal interventional practice.

The successful management of this case demonstrates, from a technical perspective, the effective integration of precise pathway control, combined device utilization, and real-time imaging guidance in the retrieval of intracardiac foreign bodies in a preterm infant. Clinically, it provides a minimally invasive alternative for managing complex catheter-related complications in high-risk neonatal populations and offers a potential reference for future procedural standardization. However, it should be acknowledged that this report represents a single-case experience, and its generalizability remains limited. In addition, the postoperative follow-up duration was restricted to one week, which should be recognized as a limitation. Consequently, potential delayed complications, including late thrombosis, arrhythmia, or subtle long-term cardiovascular functional changes, could not be comprehensively evaluated. Furthermore, the localization and retrieval of intracardiac foreign bodies require substantial interventional expertise and specialized equipment, which may limit widespread implementation in primary care settings. Future studies should focus on larger case series, procedural refinement, and extended follow-up to better define long-term safety and efficacy.

## 4 Conclusion

In summary, for emergency situations involving catheter fracture and migration into the cardiac chambers of extremely premature infants, percutaneous intravascular retrieval under fluoroscopic guidance using a DSA system without contrast represents a feasible and safe therapeutic option in selected cases. This approach may be particularly advantageous for patients with high surgical risk or contraindications to contrast media. With appropriate multidisciplinary assessment, meticulous intraoperative technique, and structured postoperative surveillance, catheter-related morbidity in neonates may be reduced. Further studies involving larger case series and longer follow-up are required to validate the safety, reproducibility, and broader applicability of this approach.

## Data Availability

The original contributions presented in the study are included in the article/supplementary material, further inquiries can be directed to the corresponding author.
